# MechanoBase: a comprehensive database for the mechanics of tissues and cells

**DOI:** 10.1093/database/baae040

**Published:** 2024-05-28

**Authors:** Yanhong Xiong, Shiyu Li, Yuxuan Zhang, Qianqian Chen, Mengtan Xing, Yong Zhang, Qi Wang

**Affiliations:** Institute for Regenerative Medicine, Shanghai East Hospital, Shanghai Key Laboratory of Signaling and Disease Research, School of Life Sciences and Technology, Tongji University, Shanghai 200092, China; Frontier Science Center for Stem Cell Research, Tongji University, Shanghai 200092, China; Institute for Regenerative Medicine, Shanghai East Hospital, Shanghai Key Laboratory of Signaling and Disease Research, School of Life Sciences and Technology, Tongji University, Shanghai 200092, China; Frontier Science Center for Stem Cell Research, Tongji University, Shanghai 200092, China; Institute for Regenerative Medicine, Shanghai East Hospital, Shanghai Key Laboratory of Signaling and Disease Research, School of Life Sciences and Technology, Tongji University, Shanghai 200092, China; Frontier Science Center for Stem Cell Research, Tongji University, Shanghai 200092, China; Institute for Regenerative Medicine, Shanghai East Hospital, Shanghai Key Laboratory of Signaling and Disease Research, School of Life Sciences and Technology, Tongji University, Shanghai 200092, China; Frontier Science Center for Stem Cell Research, Tongji University, Shanghai 200092, China; Institute for Regenerative Medicine, Shanghai East Hospital, Shanghai Key Laboratory of Signaling and Disease Research, School of Life Sciences and Technology, Tongji University, Shanghai 200092, China; Frontier Science Center for Stem Cell Research, Tongji University, Shanghai 200092, China; Institute for Regenerative Medicine, Shanghai East Hospital, Shanghai Key Laboratory of Signaling and Disease Research, School of Life Sciences and Technology, Tongji University, Shanghai 200092, China; Frontier Science Center for Stem Cell Research, Tongji University, Shanghai 200092, China; Institute for Regenerative Medicine, Shanghai East Hospital, Shanghai Key Laboratory of Signaling and Disease Research, School of Life Sciences and Technology, Tongji University, Shanghai 200092, China; Frontier Science Center for Stem Cell Research, Tongji University, Shanghai 200092, China

## Abstract

Mechanical aspects of tissues and cells critically influence a myriad of biological processes and can substantially alter the course of diverse diseases. The emergence of diverse methodologies adapted from physical science now permits the precise quantification of the cellular forces and the mechanical properties of tissues and cells. Despite the rising interest in tissue and cellular mechanics across fields like biology, bioengineering and medicine, there remains a noticeable absence of a comprehensive and readily accessible repository of this pertinent information. To fill this gap, we present MechanoBase, a comprehensive tissue and cellular mechanics database, curating 57 480 records from 5634 PubMed articles. The records archived in MechanoBase encompass a range of mechanical properties and forces, such as modulus and tractions, which have been measured utilizing various technical approaches. These measurements span hundreds of biosamples across more than 400 species studied under diverse conditions. Aiming for broad applicability, we design MechanoBase with user-friendly search, browsing and data download features, making it a versatile tool for exploring biomechanical attributes in various biological contexts. Moreover, we add complementary resources, including the principles of popular techniques, the concepts of mechanobiology terms and the cellular and tissue-level expression of related genes, offering scientists unprecedented access to a wealth of knowledge in this field of research.

**Database URL**: https://zhanglab-web.tongji.edu.cn/mechanobase/ and https://compbio-zhanglab.org/mechanobase/

## Introduction

The mechanics of tissues and cells play a crucial role in the intricate dynamics of biological systems, serving as the driving forces that orchestrate multifaceted interactions and regulate the complexity of life (1). Mechanical phenotypes, predominantly stiffness and viscosity, are determined by nuclear mechanics, cytoskeletal organization and components of the extracellular matrix (ECM) (2, 3). While providing structural support, these mechanical phenotypes can also serve as indicators of cellular or tissue health and function (4). Meanwhile, cells exist in a state of continuous exposure to various mechanical forces generated by neighboring cells and their three-dimensional microenvironment, like compression, tension and shear stress. Integral to transducing these forces is mechanosensing, whereby cells utilize an array of membrane mechanoreceptors to detect mechanical stimuli. For instance, integrins are transmembrane receptors that connect the ECM to the cytoskeleton through a complex network of adapter proteins at the focal adhesions. This detection enables the transmission of mechanical signals to the cell interior, activating signaling cascades involving pathways like Hippo, MAPK, and Rho/ROCK. These passways translate mechanical cues into consequentially biological responses, such as alterations in gene expression, cytoskeleton reorganization, and changes in mechanical properties (5–7). This underlines the significant role of mechanotransduction in governing activities and behaviors at the cellular and tissue levels, such as cell proliferation, adhesion, migration, differentiation, tissue morphogenesis, homeostasis and disease progression (8–12).

Insights derived from the mechanics of tissues and cells have influenced many fields, ranging from bioengineering to medical diagnostics. Based on the understanding of cellular responses to mechanical environments, scientists have developed biomimetic tissues and organs, advancing the field of regenerative medicine and tissue engineering (13). Furthermore, unique mechanical phenotypes have enabled novel non-invasive diagnostic tools for early disease detection (14), and therapies targeting the altered mechanical properties of malignant cells enhance treatment efficacy in oncology (15). Given the crucial functions of cellular and tissue mechanics, research in decoding life’s complexities and shaping therapeutic innovations, accurate mechanical assessment in cells and tissues has emerged as an essential area of study. Mechanical assessments involve applying a known physical force or stress to biological samples and quantifying the subsequent deformation or strain, revealing the mechanical characteristics of the examined material. Techniques such as atomic force microscopy (AFM) (16), micropipette aspiration (MA) (17) and magnetic twisting cytometry (18) provide nanoscale precision in measuring a cell’s material properties. Additionally, approaches like traction force microscopy (TFM) measure the forces that cells exert on their surrounding environment by tracking the displacement of embedded beads in the substrate (19). To assess tissue mechanics, traditional mechanical testing methods such as tensile (20), compression (21) and indentation testing (22), along with ultrasound-based techniques like elastography (23) are commonly employed. Traditional tests yield fundamental data on the tissue’s strength, elasticity and compressibility. At the same time, ultrasound-based methods evaluate tissue stiffness non-invasively by analyzing the propagation speed and pattern of transmitting waves, offering a significant contribution to *in vivo* mechanical property analysis.

The substantial surge in mechanical data underpins a remarkable expansion of the dynamic field of mechanobiology. This wealth of data facilitates research exploring the mechanical aspects of biological organisms’ structure and function, understanding the fundamental life principles, optimizing cutting-edge research equipment and developing advanced diagnosing approaches. However, the rapid accumulation of mechanobiological data brings challenges in this field due to the heterogeneity of the targeted mechanical properties, the diversity in measurement principles employed, and the variance in samples and in integrating and interpreting these disparate data sets. So far, there is no systematic repository for collecting, categorizing and managing mechanobiological data to simplify access, comparison and analysis, except for valuable resources like MechanoProDB (24), a database dedicated to exploring the mechanical properties of proteins. Here, we present MechanoBase, a comprehensive, user-friendly database that not merely archives but meaningfully categorizes this burgeoning wealth of mechanobiological data.

## Methods

### Collection of candidate articles

We conducted a comprehensive search of the PubMed database to find relevant research articles published until December 2022, using the following keywords: ‘mechanical property’, ‘mechanical characterization’, ‘mechanical force’, ‘traction’, ‘modulus’, ‘elasticity’, ‘viscosity’, ‘adhesion’ and ‘stiffness’. The Bio.Entrez package was used to collect detailed metadata from the 393 384 articles found, which included the identifiers (PMID and PMCID), title, authors, journal, date, abstract and MeSH term. In order to efficiently filter out the large volume of non-contributory results, we first filtered out approximately 119 000 articles after excluding articles that did not fit the following MeSH categories: ‘Biomechanical Phenomena’, ‘Mechanical Phenomena’, ‘Stress, Mechanical’, ‘Elasticity’, ‘Elastic Modulus’, ‘Viscosity’, ‘Elasticity Imaging Techniques’, ‘Microscopy, Atomic Force’, ‘Hardness’, ‘Tensile Strength’, ‘Shear Strength’, ‘Compressive Strength’, ‘Extracellular Matrix’ and ‘Mechanotransduction, Cellular’. Next, we created a binary classifier utilizing the pre-trained BERT (Bidirectional Encoder Representations from Transformers) multilingual base model (25). Our training data was composed of 400 carefully labeled abstracts, equally divided between Class 1 (informative) and Class 0 (non-informative, lacking keywords as mentioned). We then fine-tuned the BERT model using this training data, adjusting the model’s parameters to accurately differentiate between informative and non-informative abstracts, and resulting in a classifier optimized for our task. Finally, we applied this trained classifier to label remaining abstracts and got 26 512 candidate articles.

### Data curation

We used the PubMed Central (PMC) Open Access Service to download the full content of the candidate articles from the PMC Open Access Subset. This process provided us with a tar file for each research article, encompassing the full text in XML and PDF (if present) formats, alongside media files, tables and supplemental materials. To expedite the review process, we employed regular expressions to identify sentences that referenced measured mechanical data. We also manually documented related data, such as the organism, biological sample, method and special conditions. The majority of the data was presented as either mean ± standard deviation/standard error of the mean for normally distributed data or median (interquartile range) for non-normally distributed data. We recorded data ranges as well if provided. For articles lacking measured data, we estimated the values based on the figures provided in the articles and flagged these values with a tilde prefix.

### RNA-seq data collection and processing

Processed RNA-seq data for human tissues were directly downloaded from the Genotype-Tissue Expression (GTEx) project (26) and The Cancer Genome Atlas (TCGA) Program (https://www.cancer.gov/tcga). RNA-seq datasets for human cell lines curated in the MechanoBase were searched and downloaded from the NCBI Gene Expression Omnibus (GEO) database. Raw sequenced reads in FASTQ format were processed to trim 5ʹ low-quality bases and adaptors using Trim galore (version 0.6.10, https://www.bioinformatics.babraham.ac.uk/projects/trim_galore/) with the following parameters:—fastqc—length 40—trim_n—no_report_file—suppress_warn—paired. The trimmed RNA-seq reads were mapped to the human genome hg38 using HISAT2 (version 2.2.1) with default parameters (27). The expression level of each GENCODE (v41) gene was quantified as FPKM and TPM values using StringTie (v2.1.4, run with −e and −B parameters) (28), and read count values were obtained using GFOLD (29). For inclusion in downstream analysis and online visualization, we selected datasets based on stringent criteria: only those exhibiting an overall alignment rate exceeding 85% and a correlation coefficient greater than 0.9 between biological replicates were retained. R package DESeq2 (30) was used to perform differential expression analysis on 39 pairs of human cell lines, known for their significant differences in mechanical properties (Supplementary Table S3). Genes that differentially expressed (criteria: Log_2_FC > 1.5, *P*-adj < 0.01) in at least 32 (80%) out of the 39 comparisons were defined as candidate genes with predictive potential in cell mechanics. DAVID web service (31) was used to perform functional enrichment analysis of candidate genes. Annotated single-cell RNA-seq data in H5ad format was directly downloaded from the GEO database under accession GSE251990. Scanpy (32) was used to perform the clustering analysis.

### Web interface implementation

The server runs on the Apache web server (version 2.4.57) and is constructed using the Hypertext Preprocessor (PHP) and Bootstrap 4. Data are stored and managed through the MySQL relational database. Both the server backend and frontend–backend interactions, including functions like download and submission, are developed using PHP. The web–frontend interfaces utilize hyper text markup language (HTML), cascading style sheets (CSS) and JavaScript (JS). Moreover, to enhance user experience with interactive online visualizations, we have integrated ECharts (version 5.4.2) (33).

## Results

### Database content

**Figure 1.**
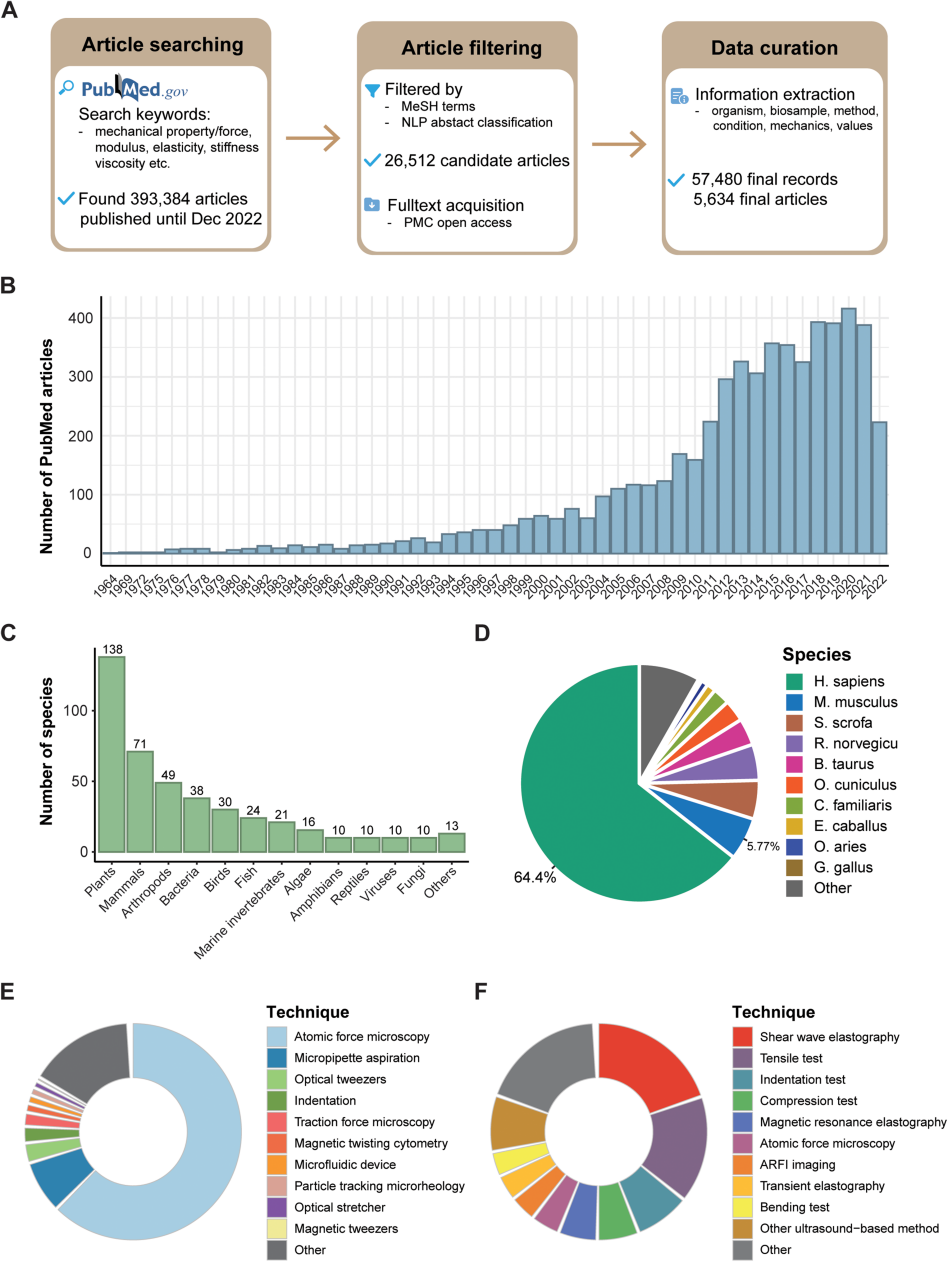
Overview of data curated in MechanoBase. (**A**) Schematic of the procedure for article search, filtration and data incorporation into MechanoBase. (**B**) Annual trend of publications related to mechanical data spanning from 1964 to 2022. (**C**) The number of species for which mechanical data have been curated, categorized by family. (**D**) Pie chart showing the distribution of mechanical data according to species. Distribution of techniques employed for assessing the mechanics of non-tissue samples (**E**) and tissue samples (**F**).

After a thorough search and a two-step filtration process of PubMed articles (see ‘Methods’ section), we identified 26 512 candidate articles published up to and including December 2022. From these, we carefully curated 57 480 records spanning 5634 articles for our MechanoBase (Figure 1A). We observed a rise in the number of such research articles over time (Figure 1B), indicating the growing research interest in this field. Although mechanical data were sourced from a broad spectrum of approximately 440 species, from viruses to mammals (Figure 1C, Supplementary Table S1), the majority of the data originated from human (64.4%) and mouse (5.77%) samples, with <30% associated with other species (Figure 1D). In our dataset, 17% of the mechanical data were non-tissue samples related, such as cells, biomacromolecules and biopolymers, and 83% were tissue samples related. For the measurements of the non-tissue samples, AFM was predominantly used (63.2%), followed by MA at 7.9% (Figure 1E). For tissue samples, ultrasound-based techniques were extensively utilized. This category includes shear wave elastography (20.5%), Acoustic Radiation Force Impulse (ARFI) imaging (4.0%), transient elastography (3.9%) and other methods (8.5%). Traditional mechanical testing methods were also significant, comprising 34.2% of the utilization followed by AFM at 4.3% (Figure 1F).

**Figure 2.**
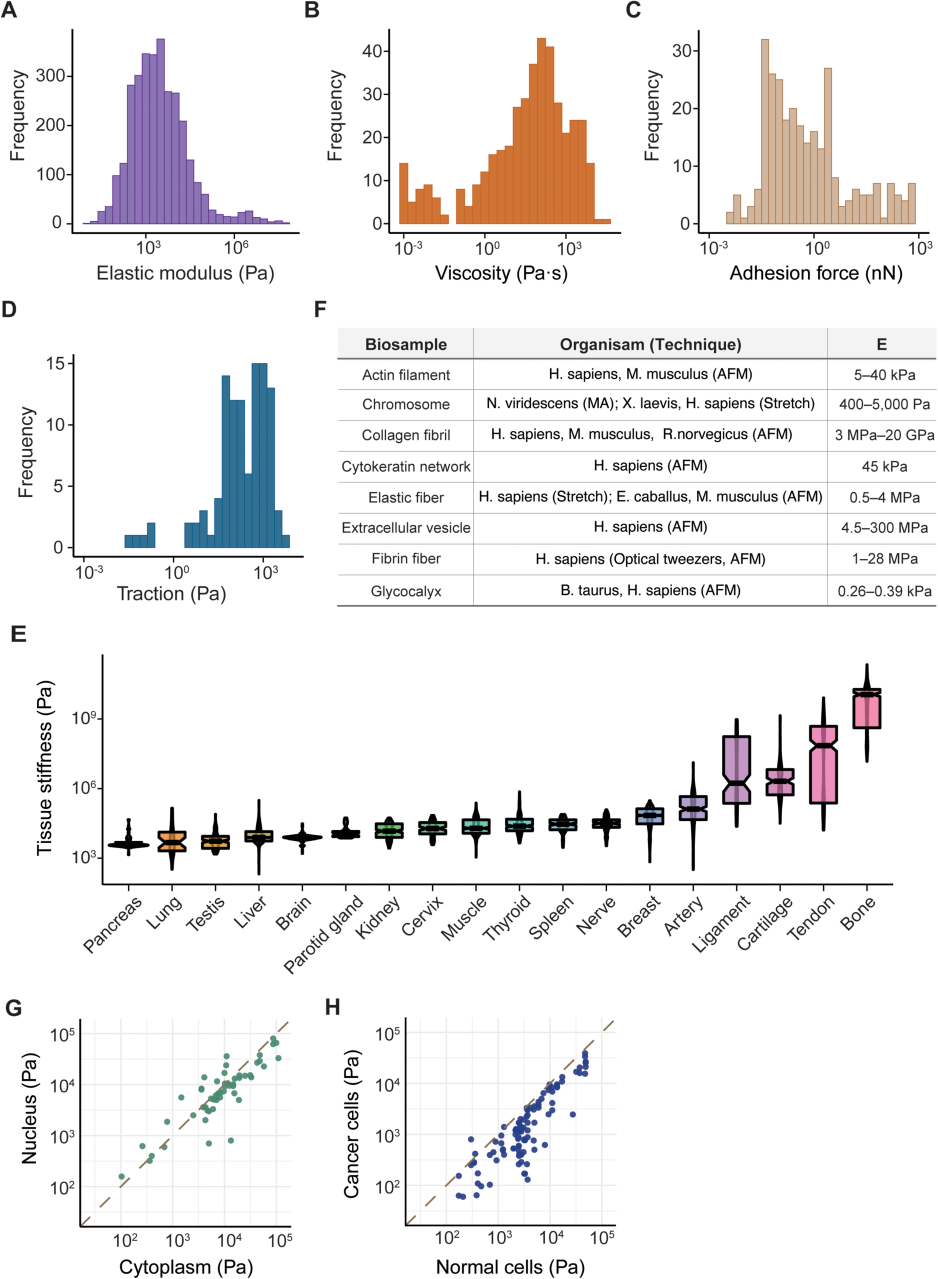
Summary of mechanical data ranged from nano- to macro-scale. Distribution of cell mechanics according to elastic modulus (**A**), viscosity (**B**), adhesion force (**C**) and traction (**D**). (**E**) Distribution of elastic modulus for 18 human tissues with the most data, boxes sorted by median value. (**F**) Table detailing various biopolymers, corresponding organisms, the techniques used for analysis and the resultant elastic modulus values. Comparison of elastic modulus between different subcellular components (**G**) and cell types (**H**). Each point represents a pair of samples from the same publication.

Elasticity refers to a biological sample’s inherent capability to revert to its original form after deformation, while viscosity describes its internal friction that resists flow or shape change. In our analysis of approximately 600 unique cell lines and types (as detailed in Supplementary Table S2), Young’s modulus, denoting their elasticity, ranged from 12.6 ± 6.1 Pa in MDA-MB-231 cells (34) to 57.56 ± 27.63 MPa in platelets (35), with a median value of 2.3 kPa (Figure 2A). Viscosity fluctuated between 0.89 ± 0.01 mPa· s in erythrocytes (36) and 34.59 ± 56.45 kPa· s in human mesenchymal stem cells (37), with a median of 73 Pa· s (Figure 2B). Our database also covered data on the generation of mechanical forces by living tissues (38, 39) and cells, including adhesion forces at the pico- to nano-Newton-scale (Figure 2C), which pertain to the amount of force required to detach the cell from the ECM (40, 41), and tractions with a median strength of 240 Pa (42, 43) (Figure 2D) generated by actomyosin contractility and transmitted to their surroundings. MechanoBase has carefully archived the material properties of hundreds of tissue samples under various conditions (Figure 2E). It also includes data on biopolymers like actin filaments (44), collagen fibrils (45–48) and fibrin fibers (49–51) (Figure 2F). A marked variation is evident in the mechanical data, spanning from nano- to macro-scale. Beyond their intrinsic properties, these values are influenced by multiple factors, like subcellular components (Figure 2G), physiological and pathological states (Figure 2H), age (52), environmental temperature (53), sample preparation and preservation (54), the technique employed (55), specific experimental parameters [AFM probe shape, loading rate, applied force (55), indentation depth (56), substrate (57), etc.] and so on. These results indicate both the comprehensiveness and the complexity of the curated data.

### Web interface

**Figure 3.**
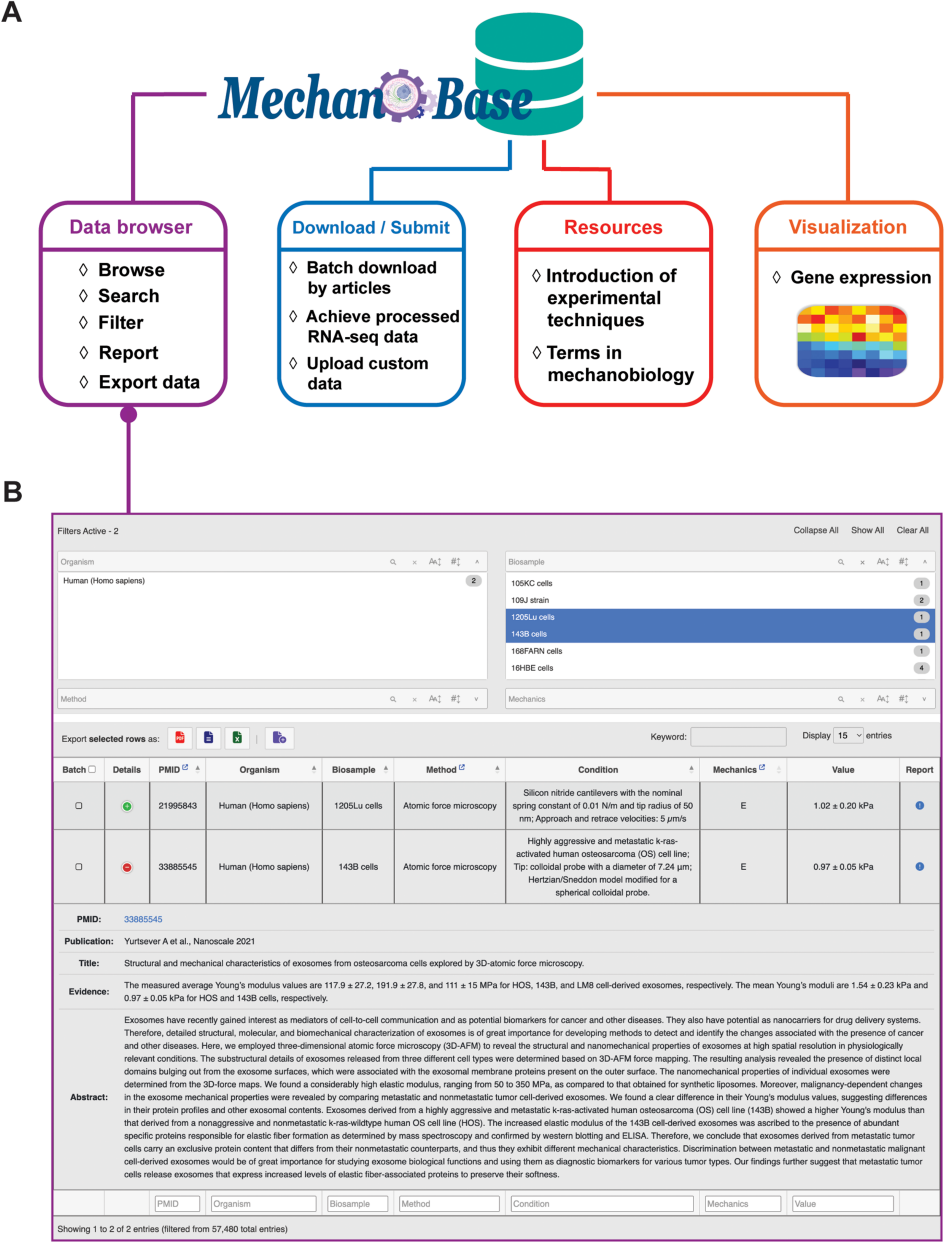
Architecture (A) and the main web application (B) of MechanoBase.

Our MechanoBase offers users various functions including data browsing, downloading/submitting, data-related techniques query and visualization (Figure 3A). As the most core function, the ‘Data browser’ component allows users to conveniently browse, search, filter, sort and download relevant datasets with detailed records related to specific organisms, biosamples, experimental methods and more, in different formats. The drop-down panes, column filtering and global filtering together facilitate multiple filtering options to precise data search. Each data row comes with a ‘Details’ button that, when clicked, gives a comprehensive view of the source article, providing users with full context. If there are inaccuracies or issues, the ‘Report’ button alongside each entry allows users to flag the problem and report the detailed incorrect information, ensuring the ongoing precision and integrity of the database (Figure 3B).

More than just a data retrieval tool, MechanoBase also allows batch download capabilities via PubMed IDs and offers processed public RNA-seq data for 95 cell lines, each accompanied by mechanical data curated within the database. Three quantification metrics—read count, FPKM and TPM—have been generated for each cell line. The ‘submission’ module allows users to upload their own data, ensuring the database stays up-to-date. The ‘Resources’ section provides essential learning materials, including overviews of experimental techniques and glossaries of mechanobiology terms.

**Figure 4.**
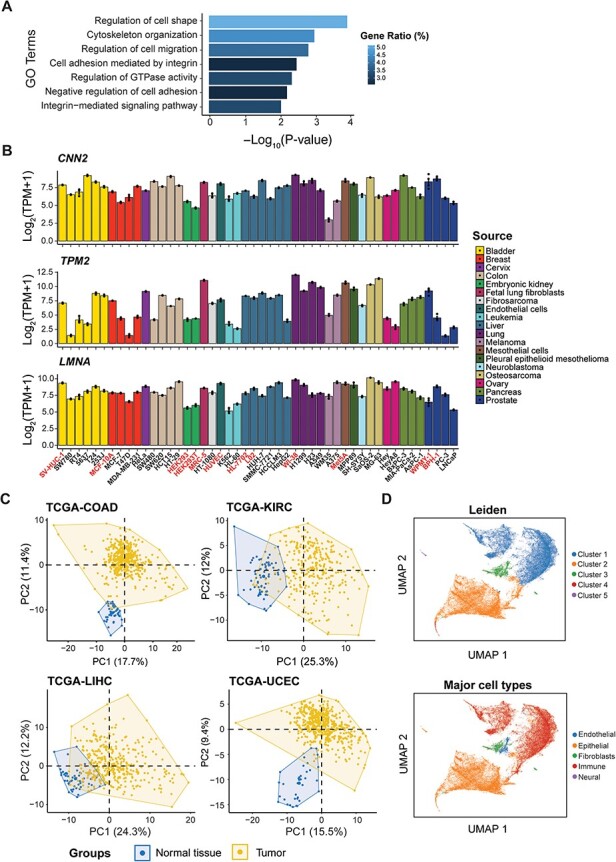
Identification of candidate genes with predictive potential in cell mechanics. (**A**) Functional enrichment analysis of the candidate genes. (**B**) The expression level of *CNN2, TPM2* and *LMNA* among cell lines from different sources used in differential expression analysis, with normal cells highlighted. (**C**) Principal component analysis (PCA) of four TCGA projects based on candidate genes. (**D**) Analysis of single-cell RNA-seq data based on candidate genes, showing clusters by Leiden (top) and major cell types from the source publication (bottom).

The ‘Visualization’ module should not be disregarded either, offering two interactive gene expression heatmap covering 155 genes indicative of cell mechanics (See ‘Methods’ section, Supplementary Table S4) across various human tissues and cells, respectively. Functional enrichment analysis revealed that these genes are predominantly involved in biological processes such as regulation of cell shape, cell migration and GTPase activity, cytoskeleton organization and cell adhesion (Figure 4A). Notably, the expression levels of these genes appear to correlate with cell elasticity. For example, the elasticity of pancreatic cancer cells was in the order of BxPC-3 > MIA-PaCa-2 > AsPC-1 (58). This order aligns with the expression patterns of genes, such as *CNN2* (Calponin 2, binds to actin filaments and is involved in the regulation of smooth muscle contraction), *TPM2* (Tropomyosin 2, an actin filament binding protein associated with the cytoskeleton and is important in controlling cell shape and mechanical properties of muscle cells) and *LMNA* (Lamin A/C, provides mechanical support to the nucleus and plays a role in gene regulation) (Figure 4B). Other important factors like *RHOB* (59), *DUSP1* (60), *CCN1* (61) and *LMO7* (62) were also included in this list. Applying our gene list to different TCGA projects resulted in a distinct separation between normal and tumor tissues (Figure 4C). Furthermore, single-cell analysis has accurately clustered cell populations that correlate well with the major cell types annotated by the source publication (63) (Figure 4D). These observations highlighted the value of mechanical gene signatures in elucidating cellular identities and characterizing their microenvironments.

### Discussion

The biomechanics of tissues and cells plays a vital role in cellular function, tissue organization and overall organismal health. The increasing mechanical data not only emphasizes the growing significance of mechanobiology in modern biomedical research but also highlights the need for structured repositories. In this paper, we have developed a pioneer, comprehensive and easy-to-use database, MechanoBase, in which all the data extracted from research papers were manually curated.

The vast spectrum of species and variety of mechanical properties catalogued in MechanoBase underscores the rich diversity inherent in biological systems. Such variability indicates the adaptive nature of different tissues and cells to their unique mechanical environments. The diverse measurement techniques used to access mechanical data, from AFM to ultrasound-based methods, offer unique perspectives. However, the diversity also brings challenges in data comparability. It is essential for researchers to consider the context and methodology when interpreting and applying the data. While MechanoBase consolidates a vast amount of data, the inherent heterogeneity across studies presents challenges in data interpretation. It becomes crucial to account for variances in sample preparation, measurement techniques and environmental conditions to derive meaningful insights. MechanoBase is indispensable for research fields like biomechanics, bioengineering and medicine. It allows researchers to investigate how cellular mechanics change under pathological and pathophysiological conditions, crucial for developing tissue development and disease progression models. It helps identify potential biomarkers for diseases with altered mechanical properties and guides drug responses. Moreover, the database can be used for simulating and predicting mechanical behaviors, as well as designing and optimizing scaffolds and biomaterials.

Incorporating RNA-seq data into our analysis provides a foundational understanding of the molecular landscape influencing mechanical behaviors. Despite challenges in aligning gene expression with mechanical properties, due to cellular heterogeneity, discrepancies in experimental conditions, and the complex nature of mechanical experiments, our work strives to bridge this gap. The data mining has unveiled genes associated with crucial mechanobiology processes, such as cytoskeleton organization and cell adhesion, reflecting cell mechanics and their mechanical microenvironment. Moreover, the relevance of protein characteristics in elucidating the biomechanical properties cannot be overstated. For example, quantification of proteins like Lamin A/C (64) and collagen (65), along with assessing the organizational structure of F-actin (66) and the subcellular localization of YAP (67) are crucial for determining the mechanical properties of cells and tissues and their responsiveness to mechanical signals. A recent study demonstrates deep learning networks predicting cellular traction forces directly from images of focal adhesion proteins like zyxin (68), advancing cell biology by connecting cellular biochemistry to physical properties. This progression suggests further studies will deepen our understanding of mechanobiology.

With the ever-increasing influx of mechanobiological data, there is a pressing need to continually refine and expand databases like MechanoBase. Future iterations could incorporate AI-driven tools to automate data extraction, conditions annotation, quality control and sophisticated analytical tools for meta-analysis. Besides, integrating more molecular-level and fluorescent image data, like metabolic or proteomic responses to mechanical changes, could offer a holistic view of mechanobiological phenomena. MechanoBase can also be extended by including mechanical properties and behaviors of biosamples predicted by computational models. These models are crucial for providing insights into scenarios where experimental data are sparse or unavailable. Last but not least, by including the mechanical properties and behaviors of artificial materials, we will enhance the database’s applicability in biomaterials research and development.

## Supplementary Material

baae040_Supp

## Data Availability

All the data in this study are collected from public databases and available at https://zhanglab-web.tongji.edu.cn/mechanobase/ and https://compbio-zhanglab.org/mechanobase/.
